# Influence of Dispersion Interactions on the Polymorphic Stability of Crystalline Oxides

**DOI:** 10.1021/acs.jpcc.3c01013

**Published:** 2023-05-26

**Authors:** Adrien Richard, Furio Corà

**Affiliations:** Department of Chemistry, University College London, 20 Gordon Street, London WC1H 0AJ, United Kingdom

## Abstract

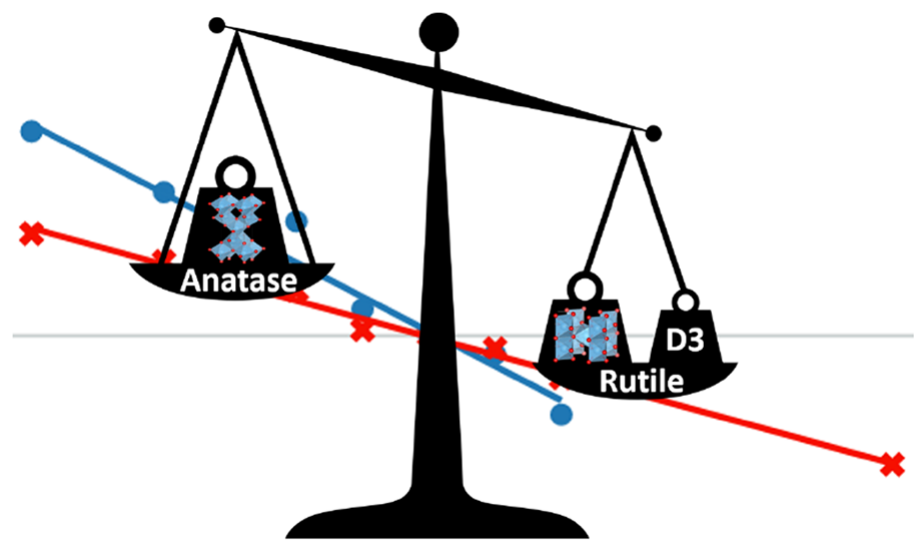

The accurate determination of relative phase stabilities using DFT methods is a significant challenge when some of these can vary by only a few kJ/mol. Here, we demonstrate that for a selection of oxides (TiO_2_, MnO_2_, and ZnO) the inclusion of dispersion interactions, accomplished using the DFT-D3 correction scheme, allows for the correct ordering and an improved calculation of the energy differences between polymorphic phases. The energetic correction provided is of the same order of magnitude as the energy difference between phases. D3-corrected hybrid functionals systematically yield results closest to experiment. We propose that the inclusion of dispersion interactions makes a significant contribution to the relative energetics of polymorphic phases, especially those with different densities, and should therefore be included for calculations of relative energies using DFT methods.

## Introduction

Density functional theory (DFT) has become a regular contributor in modern solid state chemistry, as it gives valuable insight into atomic and electronic properties of solids as well as their functional behavior, complementary to experimental results.^1,2^

The prediction of novel materials and their design requires the calculation of thermodynamic stability to a high level of precision, a need that is accentuated in materials with rich polymorphism, where differences in phase energies can be only a few kJ/mol.^3^ This has been a long-standing issue with DFT, as the choice of functional has a strong influence on the calculated formation energies and relative phase stabilities. No functional to date has produced consistent and reliable phase ordering for materials with different polymorphic forms.^4,5^ Hybrid exchange functionals (HF-DFT functionals), the current state-of-the-art for accuracy, resolve the self-interaction error (SIE) of local DFT functionals, but do not include dispersion forces.^6^ These are typically considered to be negligible in magnitude compared with the total cohesive energy of ceramic materials, dominated by Coulomb and exchange interactions. However, despite dispersion interactions representing only a small fraction of the total binding energy in solids, their energetic contribution is of the same order of magnitude as the difference in cohesive energy between different polymorphic phases.

For reliable determination of these relative phase stabilities, higher levels of theories can be employed such as second-order Møller–Plesset perturbation theory (MP2),^7^ Quantum Monte Carlo (QMC),^8^ Random Phase Approximation (RPA),^9^ or even Configuration Interaction (CI).^10^ However, these methods come at a significantly higher computational cost, which effectively limits their application to systems possessing only a few atoms. These methods also show conflicting results, with QMC-based studies predicting the correct energetic ordering only between certain TiO_2_ polymorphs, for example.^8,9^ In recent literature, SCAN (Strongly Constrained and Appropriately Normed functional) and other meta-GGA functionals^11,12^ have gained attention in relation to their successful prediction of relative phase stability in compounds, such as MnO_2_.^13^ However, they are still affected by the SIE. This can be palliated by the inclusion of empirical parameters, such as the Hubbard *U* term, but this naturally leads to a degree of empiricism and poor transferability of results.^14^

Prediction of the relative stability of TiO_2_ polymorphs is a long-standing issue for DFT calculations; this has been attributed to DFT’s inability to resolve the SIE arising from systems with localized d/f-electrons, despite the formal *d*^0^ electronic configuration of the fully oxidized Ti^4+^ cation,^4,15−19^ which makes the addition of a *U* term less justifiable. The order of stability found in the experiment,^20^ i.e., rutile < brookite < anatase is not reproduced correctly by standard GGA DFT.^21,22^ To resolve this issue, researchers have typically relied on two main solutions: the inclusion of the Hubbard *U* term or some post-SCF dispersion correction.^4,18,23−30^

The Hubbard *U* term^15,31^ introduces on-site Coulomb and exchange terms in parametric form to account for noninteger or double occupation of a subset of states such as highly localized d- and f-electron shells.^32^ A number of studies justify its use in the calculation of the relative polymorphic stability of TiO_2_ through the correction of the unphysical delocalization of Ti 3*d* electron states. Indeed, most studies using DFT+*U* report an ordering of polymorph internal energies which matches experimental findings. However, all studies recognize that the use of DFT+*U* is only a temporary solution, as not only does the inclusion of the *U* term tend to worsen the accuracy of other calculated properties, but it is also a system-specific solution.^14,33−36^

Over the past decade, DFT methods and published literature have allowed for a quantitative understanding of dispersion interactions, with the DFT-D semiempirical schemes proposed by Grimme being among the most widely applied.^37^ These treatments have shown to accurately describe the structural properties of layered materials and have proven to be especially important for calculations involving molecular crystals or low-dimensional systems, as the London dispersion interaction allows for a correct description of the intermolecular forces involved, thus producing equilibrium structures and energetic values significantly closer to experiment.^38−41^ The DFT-D schemes use damped interatomic potentials corresponding to the instantaneous dipole-induced dipole interaction so that the dispersion corrected energy becomes
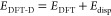
1where *E*_DFT_ is the usual mean-field DFT energy and *E*_disp_ is an empirical dispersion correction that is defined in the latest DFT-D implementation developed for solids at the time of study, DFT-D3, as the sum of two- and three-body energies, i.e., *E*_disp_ = *E*^2^ + *E*^3^. The most important two-body term is given by
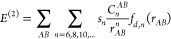
2

The first sum spans all atomic pairs in the system, while *C*_*n*_^*AB*^ represents the averaged (isotropic) *n*th-order dispersion coefficient for atom pair AB, and *r*_*AB*_ is their internuclear distance. *f*_*d*,*n*_(*r*_*AB*_) denotes the damping function applied to the energy correction. We can easily see from the representation of this sum that as *r*_*AB*_^*n*^ tends to 0, the entire sum will tend toward infinity, which will in turn theoretically infinitely increase the contribution of the two-body term toward the total dispersion correction energy *E*_DFT-D3_. *E*^3^ has a general expression of the form *C*_6_/*r*^6^ and *C*_9_(3 cos θ_*a*_ cos θ_*b*_ cos θ_*c*_ + 1)/(*r*_*AB*_*r*_*BC*_*r*_*CA*_)^3^ and is further defined in ref (40). The magnitude of the dispersion correction depends on interatomic distances and hence on the density of the studied materials.

The effect of dispersion on the formation energy of phases has been calculated and discussed in previous work,^6,42^ notably on the phase stabilities of various cesium halides.^43−45^ Similar to TiO_2_, the phase stabilities of alkali-metal halides have been a long-standing problem for DFT and their nature as simple, benchmark ionic structures spurred researchers to investigate the effects of dispersion as a computationally cheap solution to the problem. Although the importance of dispersion has been discussed for clays,^46^ metal–organic frameworks, and zeolites,^42,47^ the main focus there was on the adsorption of molecules rather than polymorphic stability. While dispersion interactions are known to be of key importance in the structural chemistry of molecular and low-dimensional crystals, they are often overlooked when considering bulk solids with strong ionic or covalent bonding. Few papers touch upon the inclusion of dispersion interactions in already established functionals as a beneficial tool toward the correct calculation of the energetic ordering of TiO_2_ polymorphs.^39,48−50^ There is no follow-up research on other compounds.

With the goal of identifying a low computational overhead method able to reproduce the polymorphic energy ordering of TiO_2_, as found in experiment, we present here a study of the relative stability of TiO_2_ polymorphs spanning a variety of functionals, which include the GGA functional PBE^51^ and the hybrid HF-DFT functionals B3LYP,^52,53^ HSE06,^54^ and PBE0,^55^ along with all their DFT-D3^40^ equivalents. The DFT-D3 correction scheme was chosen as the representative of dispersion correction methods in solids; it was shown to provide better accuracy than DFT-D2 due to the latter’s overestimation of dispersion interactions and arguably the absence of a three-body term.^6^ The study was extended to MnO_2_ and ZnO, both with a richness of crystalline polymorphs, to probe for wider applicability.

## Method

All first-principles calculations were performed using the CRYSTAL17^56^ code, in which crystalline orbitals are expanded as a linear combination of atom-centered Gaussian basis sets. For each material investigated, calculations were performed using PBE, B3LYP, HSE06, and PBE0, with and without Grimme’s D3 correction. The basis sets chosen to represent the different atomic species were all of triple valence plus polarization quality selected from the CRYSTAL Basis Set database and consistently used throughout. The basis sets used are O-8-411d1,^57^ Ti-86-411(d31),^57,58^ Mn-86-411d41G,^59^ and Zn-86-411d31G.^60^

All structures studied have been fully optimized in the space group (SG) indicated by experimental studies. CRYSTAL17^56^ default tolerances have been used for the selection of integrals, SCF convergence and geometry optimization. Reciprocal space integration has been performed via a Monkhorst–Pack mesh^61^ using an 8 × 8 x 8 grid of points for all phases.

Our calculations focus on internal energies only and do not include contributions from zero-point energy (ZPE) and vibrational entropy. All previous studies that estimated the effect of ZPE and vibrational entropy on the polymorphic phase ordering of TiO_2_ show that the impact of these effects are negligible and are unable to modify the predicted relative stability between rutile and anatase.^9,12,24,30,33−35,50,62^ ZPE and vibrational entropy effects were thus also neglected for MnO_2_ and ZnO.

For the comparison of ferromagnetic and antiferromagnetic orders in MnO_2_ polymorphs, calculations employed a broken-symmetry approach and were initiated from an ionic solution, where each Mn^4+^ ion was assigned a spin-up (α) or spin-down (β) state as required by the magnetic order studied.

## Results

### TiO_2_

#### Polymorphs

**Figure 1:**
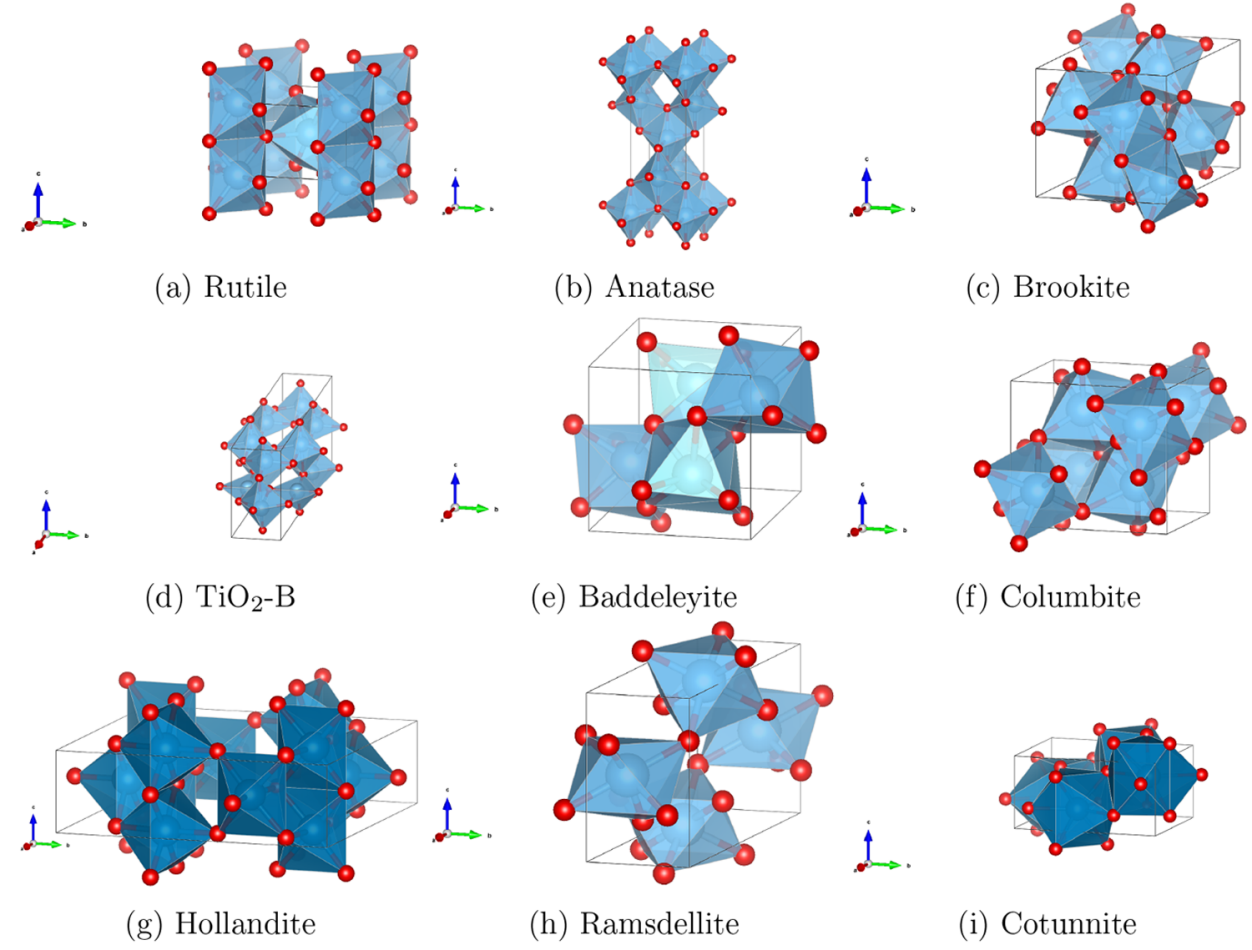
Unit cells of the different studied TiO_2_ polymorphs

We have investigated nine polymorphs of TiO_2_ whose unit cells are illustrated in Figure 1. Table 1 displays a summary of information on the Ti local environment, space group, and references for the initial structures used in our geometry optimizations. In most phases, Ti is 6-coordinated and in an octahedral (O_*h*_) environment. TiO_6_ octahedra connect in the lattices by a varied extent of corner and edge sharing, giving rise to tetragonal, orthorhombic, and monoclinic unit cells. The exceptions to the 6-coordination of Ti are baddeleyite and cotunnite, which have Ti in 7- and 9-fold coordinated environments, respectively. Rutile is known to be the ground state polymorph of TiO_2_ from experiment.^20^ The other considered polymorphs are formed at different experimental conditions according to the TiO_2_ phase diagram.^33^

**Table 1:** Space Group (SG), Ti–O Coordination Number (CN), Atomic Density (ρ) in Atoms per Cubic Ångström (a.Å^–3^) from Experiment and Reference Structural Data

	SG	CN	ρ/a.Å^–3^	ref.
rutile	*P*4_2_/*mnm*	6	0.0320	(63)
anatase	*I*4_1_/*amd*	6	0.0293	(64)
brookite	*Pbca*	6	0.0311	(65)
TiO_2_-B	*C*2/*m*	6	0.0283	(66)
columbite	*P*2_1_/*c*	6	0.0328	(67)
baddeleyite	*P*2_1_/*c*	7	0.0384	(68)
hollandite	*I*4/*m*	6	0.0313	(69)
ramsdellite	*Pnma*	6	0.0361	(70)
cotunnite	*Pnma*	9	0.0435	(71)

#### Relative Stability

**Figure 2:**
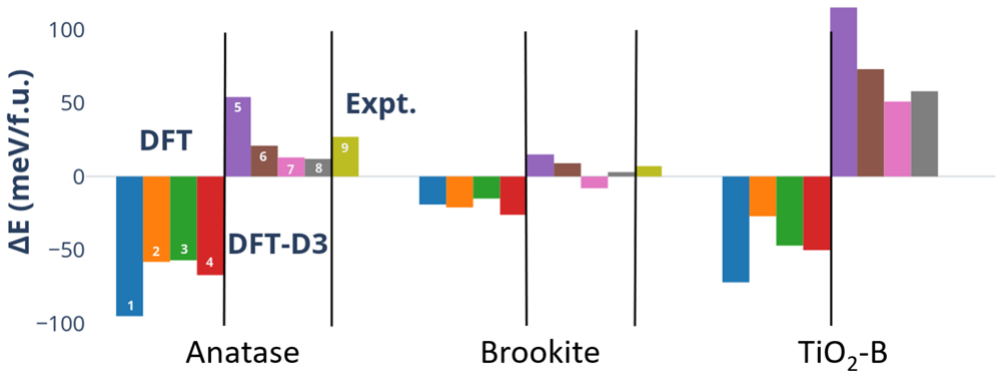
Stability of the anatase, brookite, and TiO_2_-B polymorphic phases of TiO_2_ relative to the experimental ground state rutile^20^ (Δ*E* in meV/f.u. TiO_2_) calculated with the different “standard” and dispersion-corrected functionals. Bar numbers 1–4 refer to “standard” functionals (1: B3LYP, 2: HSE06, 3: PBE, 4: PBE0), 5–8 refer to their dispersion-corrected counterparts (5: B3LYP-D3, 6: HSE06-D3, 7: PBE-D3, 8: PBE0-D3), and 9 refers to experiment where available.^20^

In this section, we focus exclusively on energetics. Structural parameters such as lattice parameters and bond distances are reported in the SI and only discussed in a global comparison of results. A collection of relative energies for all 9 TiO_2_ polymorphs from experimental and computational literature, as well as values calculated in the present work using all standard and D3-corrected functionals is provided in Table 2. All energies refer to that of rutile, the experimental ground state of TiO_2_ at ambient pressure.^20^ Different functionals provide energy estimates that differ widely not only in magnitude but also in sign, a situation that is not supportive of predictive and transferable applications. Reliable thermochemical measurements are available for only a subset of the TiO_2_ polymorphs; these are rutile, anatase and brookite^20^ i.e. the three polymorphs observed at ambient pressure. There are several calorimetry experiments providing relative stability data between these polymorphs (Table 2). Here, we consider the latest and most accurate measurements provided by Ranade et al.^20^ Of all the previous computational literature data considered, only the PBE+*U* study of ref (35) identifies rutile as the ground state, although it misrepresents the relative energy of brookite. RPA results instead find rutile to be stable over anatase, but with an energy difference of only +3.2 meV/f.u. compared to the +27.1 meV/f.u. from experiment. Results of our calculations indicate that the inclusion of dispersion through the DFT-D3 post-SCF method substantially modifies the relative stability. Indeed, while for all uncorrected functionals anatase and brookite are stable over rutile, the inverse is true upon inclusion of dispersion. In Figure 2, we examine in diagrammatic form the relative stability of the rutile, anatase, brookite and TiO_2_-B phases, grouping calculated results into standard and D3-corrected functionals. All the “standard” functionals incorrectly predict rutile as the least stable of these four polymorphs. On the other hand, all hybrid HF-DFT D3-corrected functionals (B3LYP-D3, HSE06-D3, and PBE0-D3) correctly reproduce the experimental order of phase stability.

**Table 2:** Relative Stability of TiO_2_ Polymorphs (meV/TiO_2_ Formula Unit (f.u.)) from Experimental and Computational Literature and Values Calculated in the Present Work^a^

literature	R	A	Bro.	TiO_2_-B	Col.	Bad.	H	Rams.	Cot.	ref.
expt.	0	+27.1^20^	+7.4^20,72^							(20)
expt._2_	0	+33.8^72^								(72)
Expt._3_	0	+68.1^73^								(73)
HF	0	–111.9								(4)
DFT/LDA	0	–12.1	–17.4		–20.2					(35)
B3LYP	0	–198.2								(4)
HSE06	0	–86.6	–38.3	–85.5		+122.1				(19)
PBE	0	–81.1	–40.7		–4.2	+93.6				(19,35)
PBE+*U*	0	+33.7	+35.6		+42.7					(35)
PBE-D3	0	–8.3	–15.5							(39)
PBE0	0	–61.2	–28.0							(39)
PBE0-D3	0	+18.7	+2.1							(39)
RPA	0	+3.2								(9)
DMC	0	–40.8	+0.3							(74)
SCAN	0	–25.0	–15.0	–5.3		+89.3				(19)
Current Work										
B3LYP	0	–95.1	–19.1	–72.0	–7.3	+166.5	+130.7	+122.0	+947	
HSE06	0	–58.1	–21.0	–26.8	–10.0	+93.0	+142.4	+128.1	+743.1	
PBE	0	–56.8	–15.2	–46.7	–17.5	+86.5	+120.1	+107.7	+690.7	
PBE0	0	–67.0	–26.5	–50.2	–12.8	+82.2	+135.8	+121.0	+727.9	
B3LYP-D3	0	+54.2	+15.5	+115.5	–31.6	+63.0	+397.7	+240.7	+638.1	
B3LYP-SEP-D3	0	+51.0	+29.0	+115.0	–33.0	+80.0	+404.0	+285.0	+560.0	
HSE06-D3	0	+20.7	+9.0	+73.3	–28.9	+26.1	+326.1	+221.2	+535.5	
HSE06-SEP-D3	0	+17.0	+5.0	+90.0	–22.0	+28.0	+323.0	+212.0	+560.0	
PBE-D3	0	+12.6	–7.7	+50.5	–34.3	+30.8	+253.3	+166.6	+522.2	
PBE-SEP-D3	0	+14.0	+8.0	+51.0	–30.0	+34.0	+263.0	+180.0	+532.0	
PBE0-D3	0	+11.7	+2.8	+57.6	–31.3	+23.3	+300.4	+213.9	+553.2	
PBE0-SEP-D3	0	+7.0	–1.0	+56.0	–25.0	+24.0	+296.0	+201.0	+561.0	

a Corresponding shorthand notations: rutile (R), anatase (A), brookite (Bro.), columbite (Col.), baddeleyite (Bad.), hollandite (H), ramsdellite (Rams.), and cotunnite (Cot.).

From a quantitative point of view, the HSE06-D3 functional provides the most accurate estimate of relative energy compared to experiment.^20^ Indeed, the HSE06-D3 errors are of only +2 and −6 meV/f.u. for brookite and anatase, respectively. Before corrections, the corresponding HSE06 errors are −28 and −85 meV/f.u.. The above comparison indicates that dispersion contributes as much as +87 and +42 meV/f.u to the energy of anatase and brookite relative to rutile, i.e., its quantitative influence is of the same order of magnitude as the energy difference between polymorphs and, as such, shows that dispersion interactions cannot be neglected in the prediction of the relative stability of TiO_2_ polymorphs. It is encouraging to observe that DFT-D3-corrected functionals predict the correct energy ordering for, at least, the rutile, anatase, brookite, and TiO_2_-B polymorphs. Similar quantitative effects of dispersion are observed for the HSE06, PBE, and PBE0 functionals, while for B3LYP the effect of dispersion on relative energies is even stronger. While inclusion of dispersion resolves the energetic order of the phases considered, it does still yield incorrect results for columbite. This polymorph is more stable than rutile before accounting for dispersion and inclusion of dispersion enhances the energy difference due to the higher density of columbite relative to rutile (Table 1).

In Table 2, we also include the relative stability provided by single-point energy calculations using the D3 functionals on geometries previously optimized with the equivalent standard functionals. These data are indicated with the acronym SEP-D3. The results in Table 2 indicate that the change in relative energy arises mostly from the D3 dispersion correction, while structural changes resulting from the inclusion of dispersion play only a minor role.

### MnO_2_

Many more ceramics are known to have a similar richness of polymorphic phases as TiO_2_. Here, we extend our study to MnO_2_ and ZnO compositions to verify whether the trends observed in TiO_2_ have more general validity. MnO_2_ shows polymorphism from various packings of MnO_6_ octahedra and is of interest for a variety of energy and environmental applications.^75^ There has been some amount of work on the phase ordering of MnO_2_ polymorphs, although most papers focus on a single form and all use “standard” DFT methods.^76−81^ Estimating the effect of dispersion in MnO_2_ is complicated by the additional contribution of the magnetic order, a recognized shortcoming of local DFT functionals due to the SIE, which is usually corrected through DFT+*U*. However, HF-DFT functionals also fail to accurately reproduce the energetic ordering of MnO_2_ polymorphs from experiment, despite resolving the SIE inherent to GGA DFT. Previous studies have attributed this failure to several interdependent factors such as the artificial underbinding of *O*^2–^ ligands and/or an inadequate description of exchange and correlation in the MnO_2_ polymorphs.^81^

Contrarily to TiO_2_, no research has applied methods beyond DFT to resolving the energetic ordering of MnO_2_ polymorphs, due to dispersion being considered of minor importance compared to the clarification of magnetic order. However, the SCAN functional is shown to be the method of choice to yield the correct ordering of polymorphic stability, as it is the only one to predict β-MnO_2_, the experimental ground state,^82^ as more stable than ramsdellite (R) MnO_2_.^13,83^ The only calorimetric measurements available are for the β-/R-MnO_2_ comparison.^82^ PBEsol, unlike other GGA functionals, shows the correct order of phase stability.^13,83^ References (13) and (83) also show that the inclusion of the Hubbard *U* term can cause errors in hybridization between O 2*p* and Mn 3*d* valence orbitals, resulting in unfavorable distortions of the MnO_6_ octahedra.

#### Polymorphs

**Figure 3:**
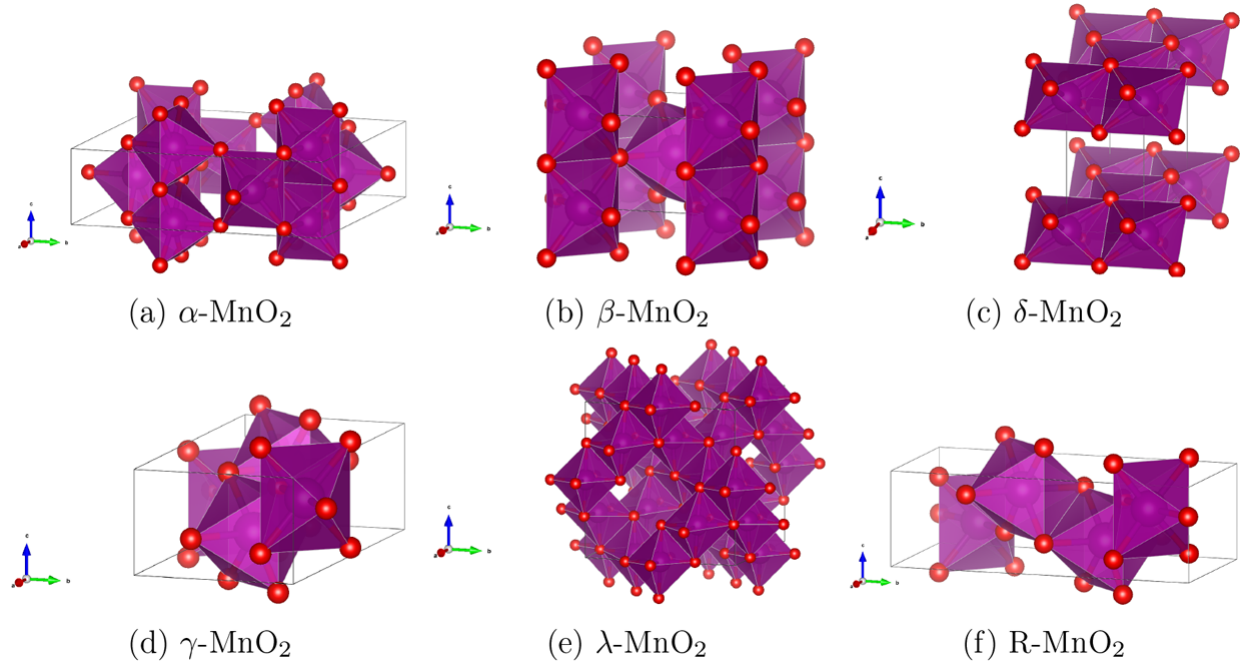
Unit cells of the different studied MnO_2_ polymorphs.

We study six polymorphs of MnO_2_ shown in Figure 3: rutile-structured pyrolusite (β-MnO_2_), the hollandite α form, the intergrowth γ form, the orthorhombic ramsdellite (R) form, the spinel λ form, and the layered δ form. Mn is 6-coordinated in all polymorphs. Table 3 displays information relating to the local environment of Mn, atomic density, space group, and references for the initial structures used in our geometry optimizations. Full structural information for the optimized phases can be found in the SI.

**Table 3:** Space Group (SG), Mn–O Coordination Number (CN), Atomic Density (ρ) in Atoms per Cubic Ångström (a.Å^–3^) from Experiment and Reference Structural Data

	SG	CN	ρ/a.Å^–3^	ref.
β	*P*4_2_/*mnm*	6	0.0359	(87)
α	*I*4/*m*	6	0.0292	(88)
δ	2/m	6	0.0321	(89)
γ	*Pnma*	6	0.0344	(90)
λ	*Fd*3*m*	6	0.0306	(91)
R	*Pnma*	6	0.0338	(70)

All calculations concerning MnO_2_ polymorphs were performed with the ground state antiferromagnetic (AFM) order, known from experiment.^13,76,84−86^ The energetic importance of magnetic coupling in MnO_2_ has been explicitly discussed in refs (13), (77), and (85−87). The energetics associated with magnetic order in MnO_2_ are greater or of the same order of magnitude as those involved with dispersion interactions and previous DFT studies have shown that choosing a ferromagnetic (FM) order yields incorrect results for lattice energies that the inclusion of dispersion corrections would be unable to palliate (Δ*E*^FM/AFM^ ∼ 150 meV/f.u. within the same polymorph using GGA functionals such as PBE).^78,81^ This was confirmed by our calculations as exemplified by Table 4.

**Table 4:** Relative Stability of MnO_2_ Polymorphs (meV/MnO_2_ f.u.) from Experimental and Computational Literature and Values Calculated in the Present Work^a^

literature	β	α	δ	γ	λ	R	ref.
expt.	0					+56	(82)
HSE06	0	–50	+30			–50	(92)
PBE	0	–40	+115	–18	+155	–35	(13)
PBE+*U*	0	–88	–5	–46	+48	–65	(13)
PBEsol	0	+30	+245	+25	+260	+20	(13)
SCAN	0	+80	+300	+45	+320	+60	(12)
current work							
B3LYP	0	–65	+32	–39	+92	–57	
HSE06	0	–62	+4	–41	+55	–62	
PBE	0	–70	+48	–35	+80	–68	
PBE0	0	–76	–22	–62	+28	–84	
B3LYP-D3	0	+84	+189	–3	+229	+7	
HSE06-D3	0	+41	+102	–20	+138	–25	
PBE-D3	0	+23	+148	–7	+160	–24	
PBE0-D3	0	+22	+76	–42	+109	–49	
Δ*E*_PBE_^FM/AFM^	105.1	83.7	7.1	90.2	8.0	92.5	

a Δ*E*_PBE_^FM/AFM^ is the energy difference (meV/MnO_2_ f.u.) between FM and AFM phases calculated with the PBE functional.

#### Relative Stability

Following the discussion on TiO_2_, we evaluate here the effect of dispersion on the energies of the MnO_2_ polymorphs. A summary of literature data and values from our study is given in Table 4. Pyrolusite (β-MnO_2_) is shown from experiment to be the ground-state polymorph^82^ and will thus be our reference for the relative stability.

All “standard” functionals find α-MnO_2_ to be more stable than the experimental ground state. However, similar to the TiO_2_ results, the incorrect ordering is reversed upon inclusion of dispersion interactions. Although “standard” PBE, HSE06, and B3LYP functionals correctly predict β-MnO_2_ to be stable over δ-MnO_2_, their D3-corrected counterparts yield a significantly larger energy difference. Both sets of D3-corrected results for the α- and δ-MnO_2_ polymorphs match experimental observations.^82^ Results are incorrect when considering R-MnO_2_, with the exception of B3LYP-D3. R-MnO_2_ is calculated as stable relative to β-MnO_2_, even after inclusion of dispersion. The energy difference, however, is substantially improved by including dispersion.

Similar to TiO_2_, in MnO_2_ the energy contribution of dispersion interactions is also of the same order of magnitude as the energetic difference between polymorphic phases and contributes to a much improved estimate of relative energies. The case of R-MnO_2_ may be influenced by the more complex crystal structure that contains two nonequivalent O sites, pyramidal and planar O, both with a CN of 3 but differentiated by their different bonding angles. The more distorted topological connectivity of O_pyr_, and thus its different relation to Mn cations compared to O_plan_, may provide an enhanced stabilization of electrostatic nature paired with the structural complexity leading to magnetic coupling interactions that are misrepresented by DFT, even when using hybrid exchange functionals. Similar arguments have been proposed in refs (13), (78), and (93). The results for columbite TiO_2_ and R-MnO_2_ thus highlight that the inclusion of dispersion interactions needs to be paired with an already accurate estimation of polymorphic stability to have an impact on results, especially when energetic contributions of the same magnitude or larger, such as magnetic coupling or change in coordination environment of some of the ions, may affect relative stabilities.

### ZnO

#### Studied Polymorphs

**Figure 4:**
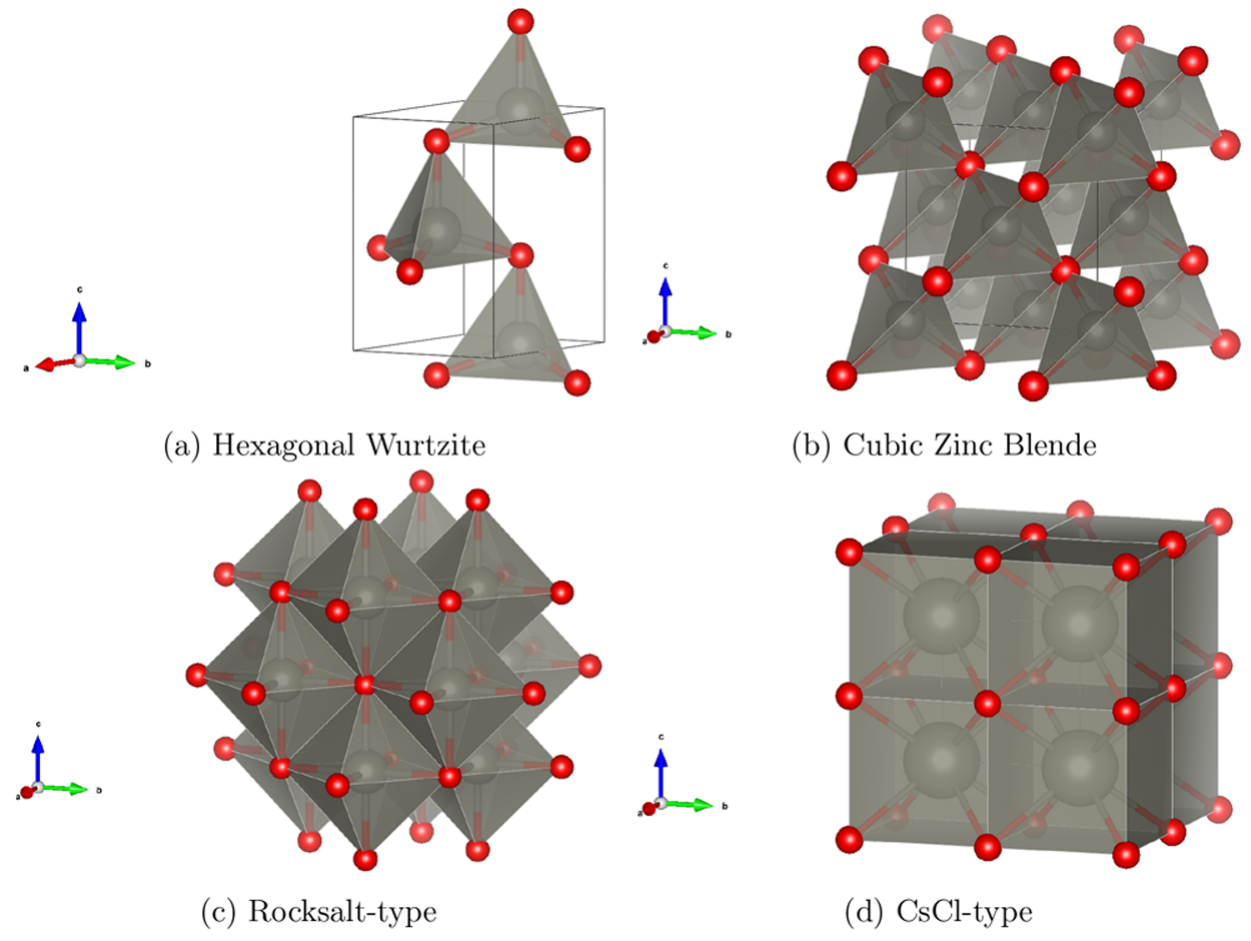
Unit cells of the different ZnO polymorphs studied.

The third system we consider is ZnO, of interest among other applications as a photocatalyst.^93^ ZnO crystallizes as hexagonal wurtzite (HW) and cubic zinc-blende (ZB) at standard conditions, with the former being the ground state.^94^ We also include, for comparison, two high pressure phases, rocksalt-type (RS) and CsCl-type ZnO shown in Figure 4. Table 5 displays structural information relating to the ZnO polymorphs studied here. Full structural information on the optimized structures is provided in the SI. Studies on the relative stability of ZnO polymorphs include DMC^95^ and RPA^96^ calculations as well as “standard” DFT-GGA techniques.^97−99^ All calculations find wurtzite as the ground state, however the relative energy of rocksalt-type ZnO is largely overestimated, and there is as yet no estimate of dispersion contribution on polymorph stability. This is an interesting topic to examine as, unlike MnO_2_ polymorphs where Mn is always in an octahedral environment, in ZnO the CN of Zn changes from 4 to 6 and 8 in the phases studied.

**Table 5:** Space Group (SG), local Zn–O Coordination Number (CN), Atomic Density in (ρ) in Atoms per Cubic Ångström (a.Å^–3^) from Experiment and Reference Structural Data

	SG	CN	ρ/a.Å^–3^	ref.
hexagonal wurtzite	*P*6_3_*mc*	4	0.0420	(100)
cubic zinc-blende	*F*43*m*	4	0.0421	(101)
rocksalt-type	*Fm*3*m*	6	0.0505	(102)
CsCl-type	*Pm*3*m*	8	0.0510	(103)

#### Relative Stability

A summary of relative energies from literature and the present study is presented in Table 6. Similar to TiO_2_, there are several calorimetry experiments evaluating the relative stability of the HW and RS polymorphs. The reference value used for comparison to experiment here is given by Sharikov et al.,^104^ as previous studies^105^ erroneously neglect the kinetic features of the HW to RS phase transition below 1000 K.

**Table 6:** Relative Stability of ZnO Polymorphs (meV/ZnO f.u.) from Experimental and Computational Literature and Values Calculated in the Present Work

literature	HW	ZB	RS	CsCl-type	ref.
expt.	0		+121.3,^104^ + 253.9^105^		(104,105)
HF	0	+57.0	+242.0	+1555.0	(106)
LDA	0	+15.0	+201.0	+4438.0	(107)
PBE	0	+12.8	+292.4	+1423.9	(108)
DMC	0	+100.0	+230.0		(95)
RPA	0	+20.0	+239.0		(96)
current work					
B3LYP	0	+26.9	+352.3	+1655.9	
HSE06	0	+23.5	+199.9	+1394.2	
PBE	0	+11.5	+247.1	+1236.3	
PBE0	0	+22.8	+197.8	+1402.2	
B3LYP-D3	0	+17.2	+172.5	+1423.4	
HSE06-D3	0	+23.6	+82.3	+1246.1	
PBE-D3	0	+10.7	+148.1	+1106.0	
PBE0-D3	0	+22.4	+88.1	+1265.7	

All functionals find HW stable over ZB and the effect of dispersion is negligible in the energy difference between these two phases, which are in practice just different stacking motifs of the same local Zn environment (also highlighted by the polymorphs’ close to identical atomic densities). The effect of dispersion is instead pronounced (over −100 meV/f.u. for B3LYP, PBE, and PBE0) when considering the relative stability of the RS polymorph, that is denser than wurtzite thanks to its higher CN. Uncorrected DFT/HF-DFT results largely overestimate the calculated calorimetric value, while after the inclusion of dispersion, the HW-RS energy difference is significantly closer to experiment, despite showing a large dependence on the functional. Once more, dispersion has similar magnitude as the energy difference between polymorphs (both of the order of 100 meV/f.u.) and must thus be accounted for in calculated thermochemistry.

### Structural Analysis

**Figure 5:**
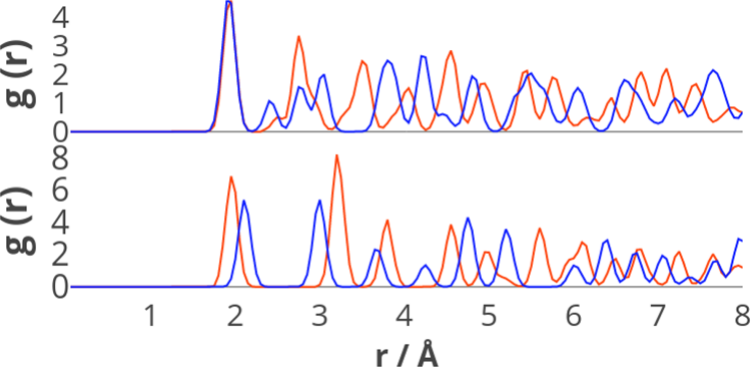
RDFs of rutile and anatase TiO_2_ (top) and hexagonal wurtzite and rocksalt-type ZnO (bottom) computed with the HSE06 functional. The red lines represent rutile TiO_2_ and HW ZnO, respectively. The blue lines represent the anatase TiO_2_ and RS ZnO, respectively.

A solid structure’s local atomic environment will have the strongest impact on the absolute value of dispersion forces, notably due to D3 dispersion coefficients being determined by an ion’s coordination number and the largest contributions occurring at small interatomic distances *r* with dispersion forces scaling as 1/*r*^6^. The distribution of bond distances in the crystal lattice can be monitored through the pair radial distribution function *g*(*r*) (RDF). The RDFs for two pairs of phases whose relative energy is affected by dispersion, i.e., rutile/anatase TiO_2_ and HW/RS ZnO, are compared in Figure 5. As we can see from Figure 5, there are noticeable changes in the local environment between the polymorph pairs, including in the short-range environments (between 2 and 4 Å). Rutile and anatase phases of TiO_2_ both have Ti in 6-fold coordination and O_*h*_ environment. The Ti–O bond distances show negligible changes in the RDF; however the phases are differentiated by next neighbor relations. The O–O distances within the same octahedron over the 2–3 Å range differ between rutile and anatase, due to the more distorted nature of the latter. This is exemplified by the three peaks at 2.4, 2.8, and 3.0 Å for anatase compared to the single, high amplitude peak at 2.75 Å for rutile. The Ti–Ti next nearest neighbor distances visible in the peaks between 3 and 4 Å suggest shorter next nearest neighbor bond distances overall in rutile.

As the stabilizing contribution to the energy given by dispersion scales as 1/*r*^6^, rutile’s more contracted local environment explains why this phase is stabilized over anatase when such dispersion forces are accounted for. In the two considered ZnO phases, instead, the CN changes from 4 to 6 between the HW and RS phases. Differences in the RDF are already obvious for the nearest Zn–O distances, but also have appreciable contributions from further neighbor shells. The higher overall density of the RS phase explains its stabilization by dispersion forces relative to wurtzite.

While the RDF contains the atomic-level information necessary to rationalize the effect of dispersion on polymorph stability, discriminating the contribution from individual atomic pairs is challenging. It would be more useful to be able to employ a global property of the materials to rationalize results. Atomic density, expressed in number of atoms per unit volume, is a simple but appropriate measure of interatomic separations in crystal lattices. We have therefore investigated whether correlations exist between dispersion energy and atomic density. We first define the dispersion contribution to polymorph stability, Δ*E*_D3_, as
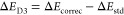
3where Δ*E*_std_ represents the relative stability between two polymorphs calculated with “standard” functionals and Δ*E*_correc_ represents the relative stability between two polymorphs calculated with D3-corrected functionals. Δ*E*_D3_ represents the sum of two contributions here i.e. the influence of the D3 correction on the single-point energy of the crystal lattice and the change in geometry stemming from the inclusion of dispersion forces through the D3 correction.

Table 7 provides a comparison of the calculated Δ*E*_D3_ for anatase relative to rutile TiO_2_ and RS relative to HW ZnO using the HSE06 functional as an example. Despite differing contributions from dispersion based on the unit cell composition, we observe a correlation between atomic density and contribution from dispersion interactions. Indeed, with a less dense cell than rutile, Δ*E*_D3_ destabilizes anatase relative to rutile, while the inverse is observed for the HW and RS ZnO polymorphs.

**Table 7:** Comparison between the Atomic Density Values in Atoms per Cubic Ångström (a.Å^–3^; from Experiment^63,64,100,102^) and the Relative Stability in meV/f.u. TiO_2_/ZnO of Both TiO_2_ (Rutile and Anatase) and ZnO (HW and RS) Polymorph Pairs Evaluated in This Section Using HSE06 and HSE06-D3 (Δ*E*_D3_)

	ρ/a.Å^–3^	Δ*E*_HSE06_	Δ*E*_HSE06-D3_	Δ*E*_D3_
rutile	0.0320			
anatase	0.0293	–58.1	+20.7	+78.8
HW	0.0420			
RS	0.0505	+199.9	+82.3	–117.6

**Figure 6:**
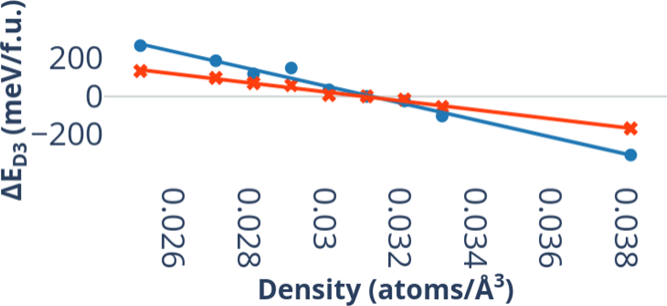
Energetic contribution from the DFT-D3 correction, Δ*E*_D3_ in meV/f.u. TiO_2_ is plotted against the atomic density in atoms per cubic Ångström (a.Å^–3^) of each TiO_2_ polymorph for the representative functionals, PBE and B3LYP. The data coloring is as follows: red for PBE and blue for B3LYP.

Figure 6 shows plots of Δ*E*_D3_ against the atomic densities for the TiO_2_ polymorphs investigated in this work, using the PBE and B3LYP functionals. Plots for other compounds (MnO_2_/ZnO) along with a complete plot for TiO_2_ can be found in the SI. In both cases there is a strong near-linear correlation, showing that the dispersion contribution increases as a function of density. The difference can be of several hundreds meV/f.u. due to a large change in density originating from a CN change as we can see for CsCl-type ZnO, for example. Smaller differences are observed between phases with the same CN and similar density, but these remain non-negligible and should therefore be included to correctly differentiate the small energy differences between these phases. We have performed a linear fit of the calculated Δ*E*_D3_ values as a function of the atomic density for each combination of composition and functional. The gradients Δ*E*_D3_^avg^ are reported in Table 8. The slope of the best fit curve is evidently compound and functional dependent, with the strongest dispersion contributions occurring in TiO_2_ and B3LYP results. PBE, PBE0, and HSE06 show comparable values.

**Table 8:** Dependence of Δ*E*_D3_ on Atomic Density from the Linear Fit of the Calculated Δ*E*_D3_ Values (Figure 6 and SI), Δ*E*_D3_^avg^ in 10^–4^ meV/Å^3^

Δ*E*_D3_^avg^·10^–4^/meV·Å^–3^	TiO_2_	MnO_2_	ZnO
B3LYP	–1.50	–0.65	–0.99
HSE06	–0.95	–0.48	–0.62
PBE	–0.79	–0.41	–0.57
PBE0	–0.86	–0.50	–0.56

The near-linear dependence on atomic density allows us to estimate the D3 correction energy as
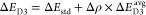
4where Δ*E*_D3_^avg^ can be obtained from the quasi-linear relationship between change in atomic density and calculated lattice energies by D3-corrected functionals (gradients of the linear fit in Figure 6 and Table 8). From its definition and the definition of its constituting parts, the calculation of Δ*E*_D3_ is evidently system-dependent and each calculated Δ*E*_D3_ value for a specific oxide will only be applicable to polymorphs of that system, as evidenced by the results in Table 8. Despite being within the same order of magnitude, we can observe some variations in calculated values of Δ*E*_D3_^avg^ between TiO_2_, MnO_2_ and ZnO.

### Discussion

Our results show that dispersion interactions are relevant to the relative stability of crystalline oxides, due to the energy contribution provided being of the same order of magnitude as the lattice energy difference between polymorphs. We have evidenced a near-linear dependence between atomic density and the energy correction provided by dispersion interactions (Figure 6 and eq 4). This is not surprising: the denser a material, the shorter its interatomic distances, *r*, resulting in a larger contribution from attractive dispersion interactions. The differences are far from inconsequential, often amounting to tens of meV/f.u..

A full list of structural parameters for the geometry optimized structures of all compositions, phases and functionals employed in the current work is provided in the SI. The number of results provided is too great to examine individually; we therefore employ a global analysis to examine the effect of dispersion on structural information. For each composition, phase and functional examined, we consider the error with respect to experiment of the three lattice parameters, equilibrium volume and shortest M–O bond distances (Δ*a*, Δ*b*, Δ*c*, δ*V*, δ*r*), respectively. The errors in each lattice parameter are then used to calculate an average error, δ*d* = . Using the equilibrium volume as an example, we define the mean and absolute errors as δ*V* =  and |δ*V*| = , respectively.

Table 9 presents the calculated errors for each “standard” and D3-corrected functional. Results presented in Table 9 are limited to phases for which an experimental structural determination at ambient pressure conditions is available and correspond to static-lattice calculations. The inclusion of the D3 contribution always causes a contraction of the equilibrium volume; for the functionals overestimating *V* (PBE), adding D3 improves the equilibrium geometry. If instead *V* is close to the experimental value or underestimated at the DFT level (HF-DFT), D3 causes excessive contraction and the equilibrium volume deviates slightly from experiment. The effect on nearest neighbor distances is negligible (δ*r* in Table 9) and only nonbonded distances are affected by the inclusion of D3. Overall, the effect of D3 on geometries is small, and the improvement yielded in relative stabilities largely outweighs any loss of accuracy on equilibrium geometry.

**Table 9:** Mean and Absolute Errors of the Different Functionals and Their D3 Counterparts Used Throughout This Study on Equilibrium Volume (δ*V*/|δ*V*|), Lattice Parameters (δ*d*/|δ*d*|) and M–O Bond Lengths (δ*r*/|δ*r*|) Compared to Experiment^63−66,70,87,88,100,102^ Over All Evaluated Materials and Polymorphs Stable at Ambient Conditions

	δ*V* (%)	δ*d* (%)	δ*r* (%)	|δ*V*| (%)	|δ*d*| (%)	|δ*r*| (%)
B3LYP	+2.90	+0.95	+1.01	2.90	0.95	1.01
HSE06	+0.20	–0.16	+0.16	0.50	0.16	0.16
PBE	+3.06	+0.05	+0.78	3.06	0.15	0.78
PBE0	+0.04	–0.61	–0.04	0.46	0.61	0.16
B3LYP-D3	–0.31	+1.01	+0.45	0.40	1.01	0.59
HSE06-D3	–1.48	+0.37	–0.04	1.48	0.37	0.58
PBE-D3	+1.10	–0.01	+0.37	1.10	0.13	0.37
PBE0-D3	–1.54	–0.63	–0.31	1.54	0.63	0.31

### Conclusions

To conclude, this work highlights the importance of the inclusion of dispersion interactions for the correct computational estimate of energetic phase orderings. This is due to both the energy differences between polymorphic phases and the dispersion interactions’ energy contribution being of tens of meV/f.u.. Due to the sparse availability of measured calorimetric data, future investigations must not only involve computational studies on an even wider range of materials to observe how distinct the influence of dispersion is in different compositions, but also new experimental work to provide a more complete and reliable experimental data set. It would also be of interest to evaluate the results obtained by DFT-D4,^109,110^ the successor to DFT-D3 used in this paper or functionals self-consistently accounting for dispersion.

## Supporting Information Available

Supporting data for all studied compounds including full structural parameter tables and remaining plots of the D3 energy as a function of polymorph density (PDF)
