# Supplementary material for: Influence of Dispersion Interactions on the Polymorphic Stability of Crystalline Oxides
Source: J Phys Chem C Nanomater Interfaces. 2023 May 26;127(22):10766–76. doi: 10.1021/acs.jpcc.3c01013 (PMC10259254; doi:10.1021/acs.jpcc.3c01013)
Supplement: Supplementary file 1 — jp3c01013_si_001.pdf [file jp3c01013_si_001.pdf]

The Influence of Dispersion Interactions on the Polymorphic Stability of Crystalline Oxides

Adrien Richard* and Furio Corà*

University College London, Department of Chemistry, University College London, 20 Gordon St, London WC1H 0AJ, London

E-mail: zccaari@ucl.ac.uk; f.cora@ucl.ac.uk

Supplementary Information

Structural Information

Structural parameter tables are in the same format for all polymorphs and compounds presenting values calculated by all standard and D3-corrected functionals as well as values from literature for lattice parameters (a, b and c in Å), unit cell volume (V in Å^3), atomic density (ρ in a.Å^{-3}) and M-O bond lengths (dependent on coordination environment in Å). Oxygen atom is in the coordination environment of the metals in each phase are labelled as in the corresponding figures.

TiO_2

- The literature values for Hollandite and Ramsdellite TiO_2 refer to MnO_2 compositions due to the unavailability of TiO_2 compositions with these unit cell structures from experiment on ICSD. Values from experiment are therefore not directly comparable to calculated values in these two cases.
- The experimental structures for the baddeleyite and cotunnite polymorphs were not obtained at ambient pressure conditions.

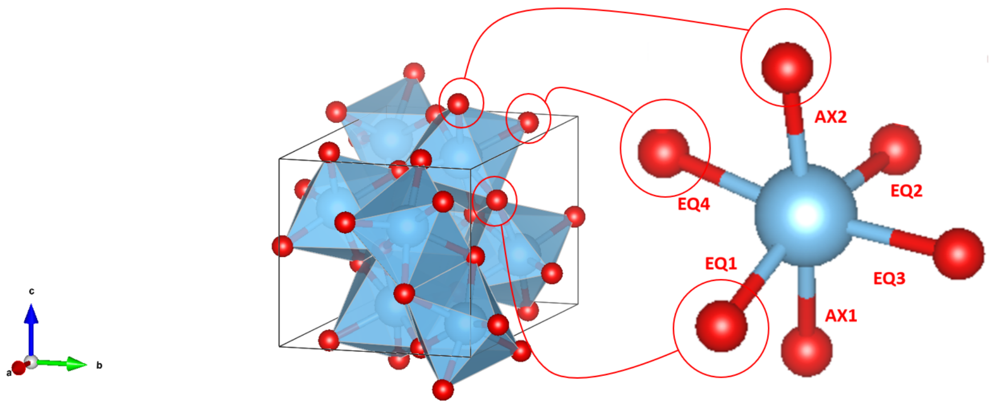

Brookite

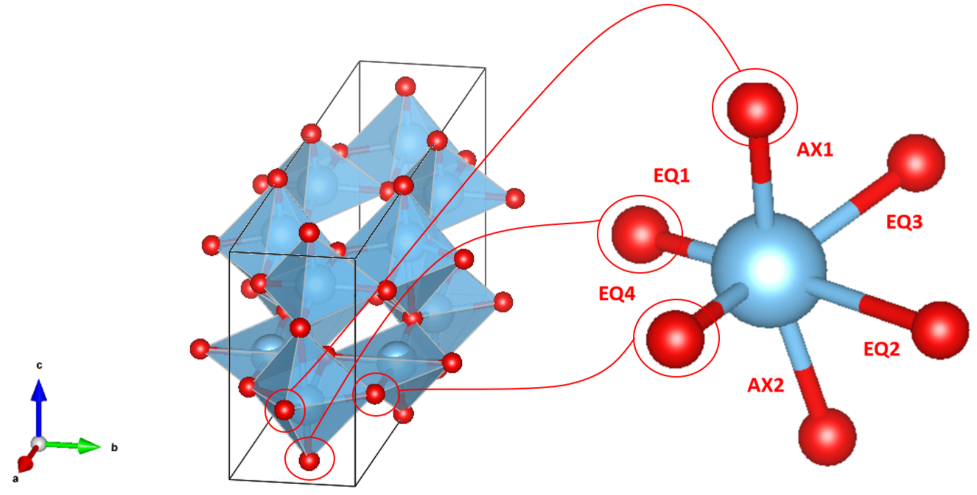

TiO_2-B

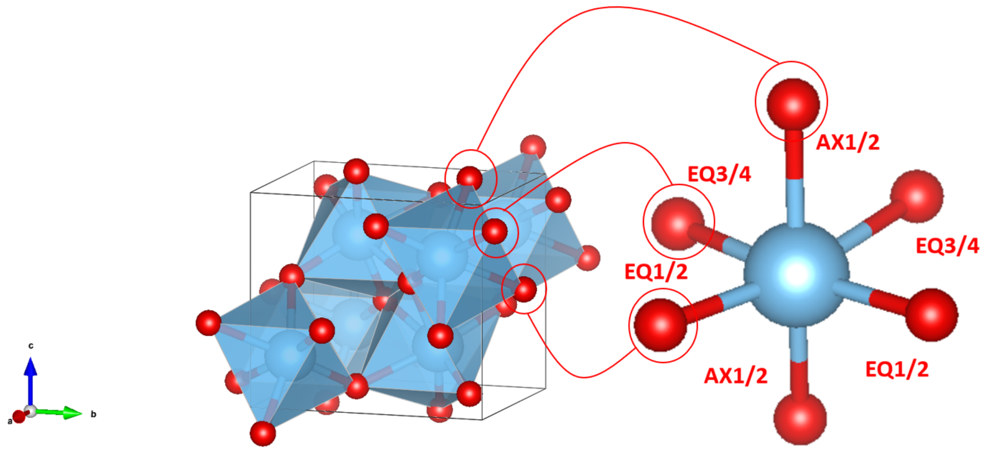

Columbite

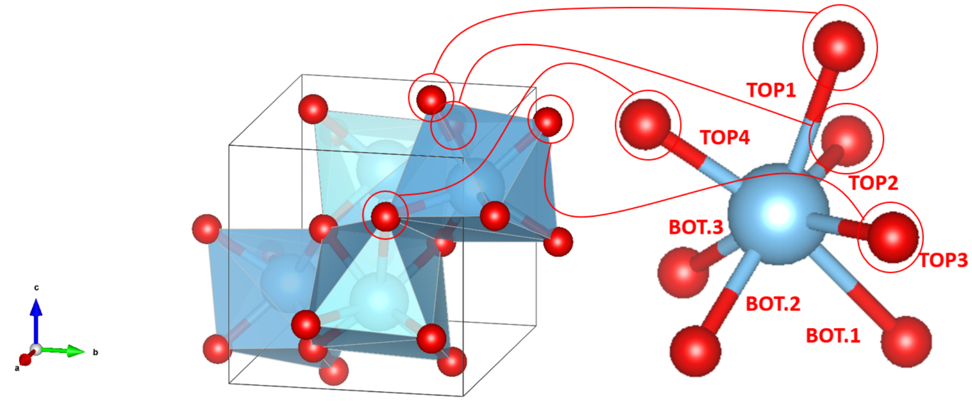

Baddeleyite

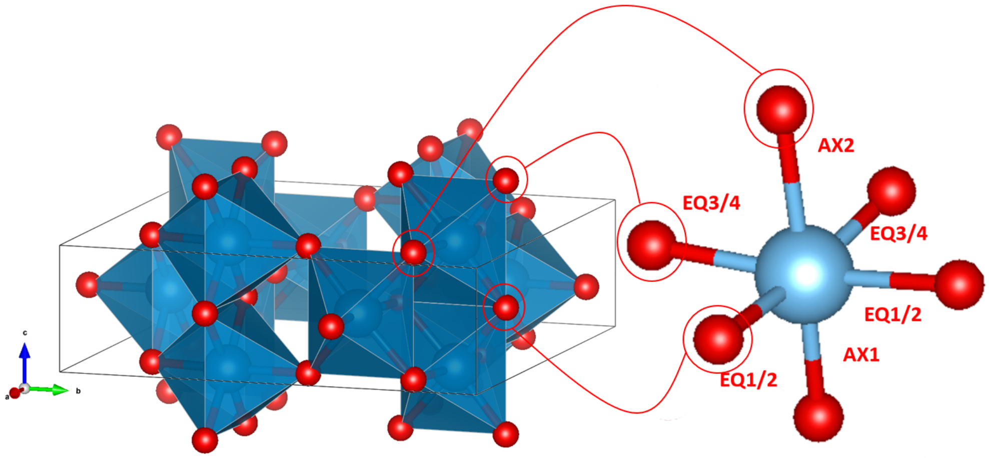

Hollandite

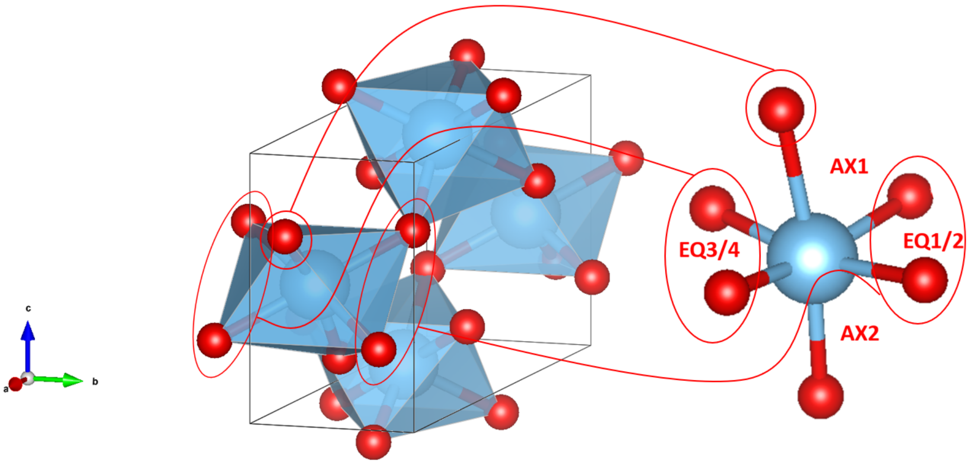

Ramsdellite

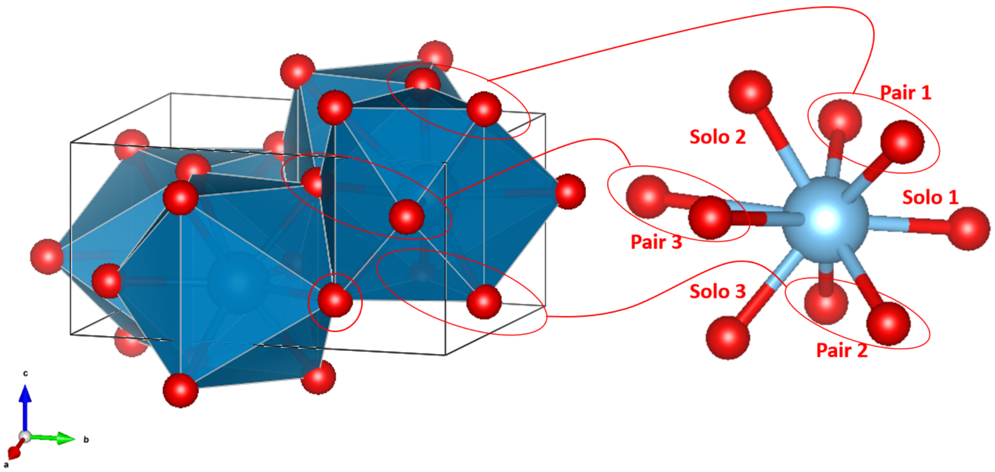

Cotunnite

Figure S1: Ti-O coordination environments of the brookite, TiO_2-B, columbite, baddeleyite, hollandite, ramsdellite and cotunnite polymorphs. Ti-O bonds are labeled according to corresponding structural parameter tables.

Rutile

Table S1: Full structural information for rutile TiO_2 from the geometry optimisations performed in this study with all evaluated functionals and their D3-corrected counterparts including: lattice parameters in Å(a, b, c), equilibrium volume in Å^3 (V), atomic density in a.Å^{-3} (ρ) and axial/equatorial Ti-O bond lengths in Å(Ti-O ax. and Ti-O eq.)

	a / Å	***b*** / Å	***c*** / Å	**V** / Å^3	ρ / **a.Å**$^{-3}$	**Ti-O ax.** / Å	**Ti-O eq.** / Å
**B3LYP**	4.626	4.626	2.981	63.81	0.0314	1.960	2.000
**HSE06**	4.587	4.587	2.963	62.34	0.0321	1.947	1.980
**PBE**	4.643	4.643	2.980	64.24	0.0311	1.964	2.003
**PBE0**	4.585	4.585	2.961	62.25	0.0321	1.945	1.980
**B3LYP-D3**	4.566	4.566	2.965	61.82	0.0324	1.948	1.966
**HSE06-D3**	4.553	4.553	2.951	61.17	0.0327	1.939	1.962
**PBE-D3**	4.609	4.609	2.970	63.10	0.0317	1.958	1.984
**PBE0-D3**	4.554	4.554	2.950	61.18	0.0327	1.938	1.963
**Expt.**[63]	4.594	4.594	2.959	62.43	0.0320	1.949	1.980

Anatase

Table S2: Full structural information for anatase TiO_2 from the geometry optimisations performed in this study with all evaluated functionals and their D3-corrected counterparts including: lattice parameters in Å(a, b, c), equilibrium volume in $Å^3$ (V), atomic density in a.$Å^{-3}$ (ρ) and axial/equatorial Ti-O bond lengths in Å(Ti-O ax. and Ti-O eq.)

	***a* / Å**	***b* / Å**	***c* / Å**	**V / $Å^3$**	**ρ / a.$Å^{-3}$**	**Ti-O ax. / Å**	**Ti-O eq. / Å**
**B3LYP**	3.792	3.792	9.759	140.31	0.0285	2.000	1.946
**HSE06**	3.772	3.772	9.633	137.04	0.0292	1.983	1.933
**PBE**	3.806	3.806	9.731	140.96	0.0284	2.006	1.950
**PBE0**	3.773	3.773	9.615	136.84	0.0292	1.980	1.933
**B3LYP-D3**	3.769	3.769	9.554	135.71	0.0295	1.983	1.928
**HSE06-D3**	3.759	3.759	9.505	134.32	0.0298	1.972	1.923
**PBE-D3**	3.795	3.795	9.602	138.29	0.0289	1.995	1.940
**PBE0-D3**	3.760	3.760	9.502	134.32	0.0298	1.970	1.923
**Expt.**[64]	3.784	3.784	9.515	136.25	0.0293	1.980	1.934

Brookite

Table S3: Full structural information for brookite TiO_2 from the geometry optimisations performed in this study with all evaluated functionals and their D3-corrected counterparts including: lattice parameters in Å(a, b, c), equilibrium volume in Å^3 (V), atomic density in a.Å^{-3} (ρ) and axial/equatorial Ti-O bond lengths in Å(Ti-O ax. and Ti-O eq.)

	a / Å	b / Å	c / Å	V / Å^3	ρ / a.Å^{-3}	Ti-O ax$_1$ / Å	Ti-O ax$_2$ / Å	Ti-O eq$_1$ / Å	Ti-O eq$_2$ / Å	Ti-O eq$_3$ / Å	Ti-O eq$_4$ / Å
**B3LYP**	9.254	5.494	5.183	263.5	0.0304	2.014	1.932	1.868	2.086	1.943	2.009
**HSE06**	9.195	5.443	5.139	257.2	0.0311	1.991	1.921	1.865	2.052	1.936	1.988
**PBE**	9.279	5.508	5.184	264.9	0.0302	2.024	1.931	1.871	2.099	1.942	2.016
**PBE0**	9.191	5.442	5.136	256.9	0.0311	1.989	1.920	1.863	2.051	1.935	1.989
**B3LYP-D3**	9.162	5.432	5.128	255.2	0.0314	2.004	1.913	1.858	2.052	1.930	1.976
**HSE06-D3**	9.137	5.409	5.105	252.3	0.0317	1.987	1.909	1.859	2.033	1.928	1.968
**PBE-D3**	9.227	5.471	5.152	260.1	0.0308	2.018	1.923	1.868	2.071	1.938	1.993
**PBE0-D3**	9.138	5.411	5.105	252.4	0.0317	1.986	1.908	1.857	2.034	1.928	1.969
**Expt.**[65]	9.174	5.449	5.138	256.84	0.0311	1.999	1.923	1.863	2.052	1.931	1.990

TiO_2–B

Table S4: Full structural information for TiO_2-B from the geometry optimisations performed in this study with all evaluated functionals and their D3-corrected counterparts including: lattice parameters in Å(a, b, c), equilibrium volume in Å^3 (V), atomic density in a.Å^{-3} (ρ) and axial/equatorial Ti-O bond lengths in Å(Ti-O ax. and Ti-O eq.)

	a / Å	b / Å	c / Å	V / Å^3	ρ / a.Å^{-3}	Ti-O ax$_1$ / Å	Ti-O ax$_2$ / Å	Ti-O eq$_1$ / Å	Ti-O eq$_2$ / Å	Ti-O eq$_3$ / Å	Ti-O eq$_4$ / Å
**B3LYP**	12.296	3.758	6.620	292.5	0.0274	2.028	1.927	1.770	2.369	1.963	1.963
**HSE06**	12.215	3.741	6.540	285.9	0.0280	2.005	1.919	1.760	2.313	1.953	1.953
**PBE**	12.324	3.770	6.620	294.2	0.0272	2.033	1.930	1.783	2.339	1.967	1.967
**PBE0**	12.198	3.740	6.540	285.4	0.0280	2.001	1.919	1.759	2.318	1.953	1.953
**B3LYP-D3**	12.193	3.721	6.558	284.4	0.0281	2.018	1.907	1.762	2.334	1.943	1.943
**HSE06-D3**	12.139	3.718	6.507	280.9	0.0285	1.998	1.903	1.755	2.298	1.942	1.942
**PBE-D3**	12.260	3.751	6.588	289.7	0.0276	2.027	1.917	1.777	2.327	1.956	1.956
**PBE0-D3**	12.139	3.719	6.507	281.0	0.0285	1.996	1.905	1.754	2.299	1.942	1.942
**Expt.**[66]	12.160	3.740	6.510	282.69	0.0283	2.067	1.727	1.681	2.374	2.003	2.003

Columbite

Table S5: Full structural information for Columbite TiO_2 from the geometry optimisations performed in this study with all evaluated functionals and their D3-corrected counterparts including: lattice parameters in Å(a, b, c), equilibrium volume in Å^3 (V), atomic density in a.Å^{-3} (ρ) and axial/equatorial Ti-O bond lengths in Å(Ti-O ax. and Ti-O eq.)

	a / Å	b / Å	c / Å	V / Å^3	ρ / a.Å^{-3}	Ti-O ax$_{1/2}$ / Å	Ti-O eq$_{1/2}$ / Å	Ti-O eq$_{3/4}$ / Å
**B3LYP**	4.578	5.550	4.942	125.6	0.0319	1.963	1.883	2.116
**HSE06**	4.549	5.487	4.905	122.4	0.0333	1.948	1.877	2.085
**PBE**	4.598	5.556	4.945	126.3	0.0317	1.967	1.886	2.124
**PBE0**	4.546	5.486	4.902	122.3	0.0327	1.947	1.876	2.086
**B3LYP-D3**	4.528	5.480	4.901	121.6	0.0329	1.948	1.871	2.083
**HSE06-D3**	4.528	5.440	4.870	120.0	0.0333	1.937	1.870	2.066
**PBE-D3**	4.573	5.511	4.920	124.0	0.0323	1.958	1.879	2.104
**PBE0-D3**	4.526	5.446	4.871	120.1	0.0333	1.938	1.869	2.068
**Expt.**[67]	4.527	5.497	4.900	122.0	0.0328	1.950	1.881	2.068

Baddeleyite

Table S6: Full structural information for baddeleyite TiO_2 from the geometry optimisations performed in this study with all evaluated functionals and their D3-corrected counterparts including: lattice parameters in Å(a, b, c), equilibrium volume in Å^3 (V), atomic density in a.Å^{-3} (ρ) and axial/equatorial Ti-O bond lengths in Å(Ti-O ax. and Ti-O eq.)

	a / Å	b / Å	c / Å	V / Å^3	ρ / a.Å^{-3}	Ti-O top$_1$ / Å	Ti-O top$_2$ / Å	Ti-O top$_3$ / Å	Ti-O top$_4$ / Å	Ti-O bot.$_1$ / Å	Ti-O bot.$_2$ / Å	Ti-O bot.$_3$ / Å
**B3LYP**	4.863	4.881	5.117	119.8	0.0334	2.181	1.990	1.985	1.876	2.282	1.976	2.047
**HSE06**	4.809	4.857	5.028	115.8	0.0345	2.128	1.999	1.996	1.876	2.190	1.953	2.022
**PBE**	4.854	4.902	5.094	119.5	0.0335	2.155	2.012	2.007	1.886	2.231	1.973	2.057
**PBE0**	4.807	4.856	5.024	115.7	0.0346	2.124	1.997	1.996	1.874	2.191	1.954	2.019
**B3LYP-D3**	4.788	4.846	5.007	114.7	0.0349	2.123	2.009	1.992	1.872	2.156	1.943	2.028
**HSE06-D3**	4.759	4.849	4.950	112.7	0.0355	2.091	2.014	1.993	1.882	2.124	1.925	2.017
**PBE-D3**	4.809	4.887	5.033	116.7	0.0343	2.125	2.025	2.004	1.885	2.171	1.953	2.050
**PBE0-D3**	4.761	4.847	4.956	112.8	0.0355	2.091	2.011	1.993	1.877	2.132	1.930	2.015
**Expt.**[68]	4.589	4.849	4.736	104.2	0.0384	2.119	1.938	1.920	1.832	2.273	1.902	1.938

Hollandite

Table S7: Full structural information for hollandite TiO_2 from the geometry optimisations performed in this study with all evaluated functionals and their D3-corrected counterparts including: lattice parameters in Å(a, b, c), equilibrium volume in $Å^3$ (V), atomic density in a.$Å^{-3}$ (ρ) and axial/equatorial Ti-O bond lengths in Å(Ti-O ax. and Ti-O eq.)

	a / Å	b / Å	c / Å	V / $Å^3$	ρ / a.$Å^{-3}$	Ti-O ax_1 / Å	Ti-O ax_2 / Å	Ti-O $eq_{1/2}$ / Å	Ti-O $eq_{3/4}$ / Å
**B3LYP**	10.300	10.300	2.993	317.5	0.0252	1.881	2.118	2.042	1.907
**HSE06**	10.193	10.193	2.969	308.5	0.0259	1.885	2.065	2.013	1.905
**PBE**	10.313	10.313	2.986	317.6	0.0252	1.885	2.127	2.045	1.912
**PBE0**	10.189	10.189	2.968	308.1	0.0260	1.884	2.066	2.013	1.903
**B3LYP-D3**	10.128	10.128	2.969	304.5	0.0263	1.861	2.096	2.021	1.903
**HSE06-D3**	10.084	10.084	2.953	300.3	0.0266	1.870	2.059	2.002	1.900
**PBE-D3**	10.205	10.205	2.973	309.6	0.0258	1.874	2.109	2.033	1.911
**PBE0-D3**	10.095	10.095	2.954	301.0	0.0266	1.872	2.057	2.002	1.900
**Expt.**[69]	9.788	9.788	2.865	274.5	0.0313	1.894	1.921	1.882	1.927

Ramsdellite

Table S8: Full structural information for ramsdellite TiO_2 from the geometry optimisations performed in this study with all evaluated functionals and their D3-corrected counterparts including: lattice parameters in Å(a, b, c), equilibrium volume in $Å^3$ (V), atomic density in a.$Å^{-3}$ (ρ) and axial/equatorial Ti-O bond lengths in Å(Ti-O ax. and Ti-O eq.)

	a / Å	b / Å	c / Å	V / $Å^3$	ρ / a.$Å^{-3}$	Ti-O ax_1 / Å	Ti-O ax_2 / Å	Ti-O $eq_{1/2}$ / Å	Ti-O $eq_{3/4}$ / Å
**B3LYP**	9.514	2.992	4.945	140.8	0.0284	2.121	1.879	2.043	1.907
**HSE06**	9.415	2.968	4.878	136.3	0.0294	2.074	1.880	2.016	1.904
**PBE**	9.470	2.985	4.955	140.0	0.0286	2.134	1.882	2.047	1.912
**PBE0**	9.401	2.966	4.877	136.0	0.0294	2.074	1.879	2.015	1.902
**B3LYP-D3**	9.196	2.966	4.738	129.2	0.0310	2.120	1.847	2.025	1.898
**HSE06-D3**	9.615	2.956	4.634	131.7	0.0304	2.004	1.900	1.976	1.921
**PBE-D3**	9.474	2.976	4.803	135.4	0.0295	2.112	1.870	2.032	1.913
**PBE0-D3**	9.617	2.956	4.638	131.9	0.0303	2.000	1.903	1.975	1.922
**Expt.**[70]	9.270	2.866	4.533	120.4	0.0361	1.889	1.910	1.862	1.923

Cotunnite

Table S9: Full structural information for cotunnite TiO_2 from the geometry optimisations performed in this study with all evaluated functionals and their D3-corrected counterparts including: lattice parameters in Å(a, b, c), equilibrium volume in Å^3 (V), atomic density in a.Å^{-3} (ρ) and axial/equatorial Ti-O bond lengths in Å(Ti-O ax. and Ti-O eq.)

	a / Å	b / Å	c / Å	V / Å^3	ρ / a.Å^{-3}	Ti-O pair$_1$ / Å	Ti-O pair$_2$ / Å	Ti-O pair$_3$ / Å	Ti-O solo$_1$ / Å	Ti-O solo$_2$ / Å	Ti-O solo$_3$ / Å
**B3LYP**	5.221	3.241	6.222	105.3	0.0380	2.094	2.099	2.620	1.955	2.040	2.047
**HSE06**	5.163	3.166	6.275	102.6	0.0390	2.066	2.071	2.576	1.977	2.022	2.050
**PBE**	5.244	3.176	6.303	105.0	0.0381	2.074	2.109	2.549	2.024	2.049	2.054
**PBE0**	5.169	3.155	6.261	102.1	0.0392	2.059	2.073	2.561	1.981	2.024	2.046
**B3LYP-D3**	5.169	3.142	6.222	101.0	0.0396	2.049	2.080	2.525	1.993	2.022	2.029
**HSE06-D3**	5.178	3.099	6.136	98.5	0.0406	2.012	2.100	2.440	2.008	2.008	2.023
**PBE-D3**	5.246	3.126	6.207	101.8	0.0393	2.037	2.127	2.453	2.023	2.045	2.047
**PBE0-D3**	5.172	3.105	6.151	98.8	0.0405	2.018	2.094	2.458	2.002	2.013	2.021
**Expt.**[71]	5.163	2.989	5.966	92.1	0.0435	1.953	2.006	2.360	1.909	2.056	2.167

MnO_2

- Lattice parameter values slightly differ for the α-MnO_2 structure compared to Hollandite TiO_2 despite them being isostructural. Different ICSD structures were used for each polymorph material[69,88].

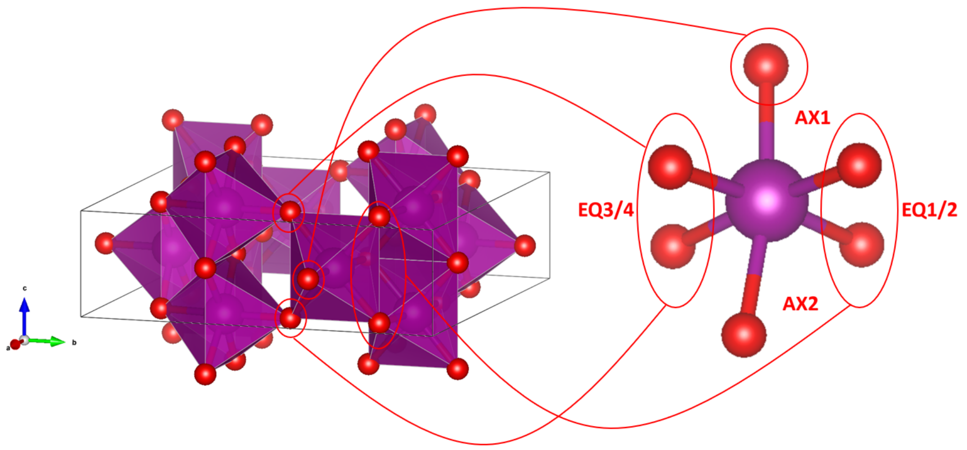

α-MnO_2

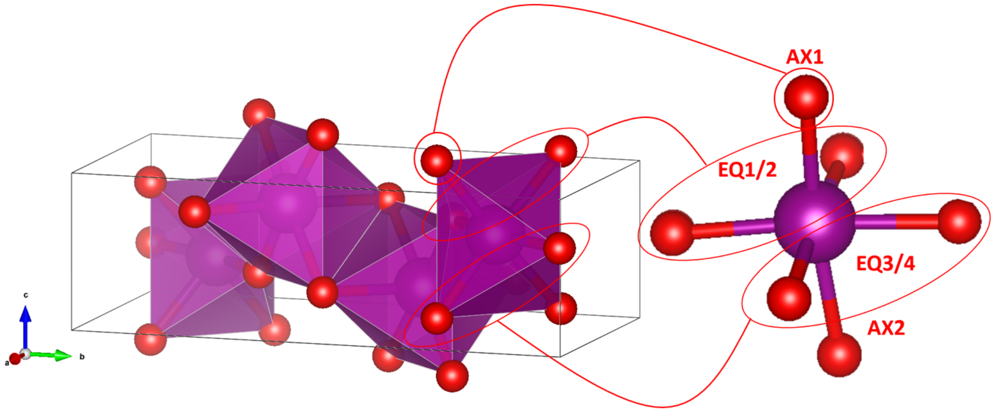

R-MnO_2

Figure S2: Mn-O coordination environments of the α-MnO_2 and R-MnO_2polymorphs. Mn-O bonds are labeled according to corresponding structural parameter tables.

β-MnO_2

Table S10: Full structural information for β-MnO_2 from the geometry optimisations performed in this study with all evaluated functionals and their D3-corrected counterparts including: lattice parameters in Å(a, b, c), equilibrium volume in Å^3 (V), atomic density in a.Å^{-3} (ρ) and axial/equatorial Mn-O bond lengths in Å(Mn-O ax. and Mn-O eq.)

	***a* / Å**	***b* / Å**	***c* / Å**	**V / Å^3**	**ρ / a.Å^{-3}**	**Mn-O ax. / Å**	**Mn-O eq. / Å**
**B3LYP**	4.430	4.430	2.895	56.8	0.0352	1.918	1.890
**HSE06**	4.385	4.385	2.876	55.3	0.0366	1.898	1.875
**PBE**	4.438	4.438	2.891	56.9	0.0351	1.913	1.895
**PBE0**	4.383	4.383	2.873	55.2	0.0362	1.898	1.873
**B3LYP-D3**	4.396	4.396	2.881	55.7	0.0359	1.900	1.880
**HSE06-D3**	4.366	4.366	2.867	54.6	0.0366	1.888	1.869
**PBE-D3**	4.418	4.418	2.881	56.2	0.0356	1.902	1.889
**PBE0-D3**	4.365	4.365	2.866	54.6	0.0366	1.888	1.868
**Expt.**[87]	4.404	4.404	2.877	55.8	0.0359	1.897	1.884

δ-MnO_2

Table S11: Full structural information for δ-MnO_2 from the geometry optimisations performed in this study with all evaluated functionals and their D3-corrected counterparts including: lattice parameters in Å(a, b, c), equilibrium volume in Å^3 (V), atomic density in a.Å^{-3} (ρ) and axial/equatorial Mn-O bond lengths in Å(Mn-O ax. and Mn-O eq.)

	***a* / Å**	***b* / Å**	***c* / Å**	**V / Å^3**	**ρ / a.Å^{-3}**	**Mn-O ax. / Å**	**Mn-O eq. / Å**
**B3LYP**	5.000	2.887	5.417	78.1	0.0256	1.916	1.916
**HSE06**	4.946	2.854	5.005	70.5	0.0284	1.901	1.899
**PBE**	4.999	2.883	5.199	74.6	0.0268	1.924	1.923
**PBE0**	4.940	2.851	4.903	69.1	0.0290	1.899	1.898
**B3LYP-D3**	4.969	2.870	4.354	62.1	0.0322	1.909	1.908
**HSE06-D3**	4.928	2.844	4.408	61.8	0.0324	1.896	1.895
**PBE-D3**	4.977	2.873	4.470	63.9	0.0313	1.920	1.919
**PBE0-D3**	4.924	2.844	4.419	61.9	0.0323	1.895	1.894
**Expt.**[89]	5.175	2.850	4.337	62.3	0.0321	1.919	1.796

γ-MnO_2

Table S12: Full structural information for γ-MnO_2 from the geometry optimisations performed in this study with all evaluated functionals and their D3-corrected counterparts including: lattice parameters in Å(a, b, c), equilibrium volume in Å^3 (V), atomic density in a.Å^{-3} (ρ) and axial/equatorial Mn-O bond lengths in Å(Mn-O ax. and Mn-O eq.)

	a / Å	b / Å	c / Å	V / Å^3	ρ / a.Å^{-3}	Mn-O ax. / Å	Mn-O eq. / Å
**B3LYP**	13.734	2.889	4.527	179.4	0.0334	1.918	1.893
**HSE06**	13.618	2.866	4.466	174.2	0.0344	1.899	1.877
**PBE**	13.834	2.886	4.488	179.1	0.0335	1.915	1.898
**PBE0**	13.605	2.864	4.465	173.8	0.0345	1.898	1.875
**B3LYP-D3**	13.766	2.873	4.405	174.1	0.0345	1.898	1.882
**HSE06-D3**	13.647	2.856	4.389	171.0	0.0351	1.888	1.870
**PBE-D3**	13.880	2.875	4.393	175.2	0.0342	1.901	1.893
**PBE0-D3**	13.630	2.854	4.393	170.8	0.0351	1.887	1.869
**Expt.**[90]	13.828	2.873	4.386	174.2	0.0344	1.905	1.886

λ-MnO_2

Table S13: Full structural information for λ-MnO_2 from the geometry optimisations performed in this study with all evaluated functionals and their D3-corrected counterparts including: lattice parameters in Å(a, b, c), equilibrium volume in Å^3 (V), atomic density in a.Å^{-3} (ρ) and axial/equatorial Mn-O bond lengths in Å(Mn-O ax. and Mn-O eq.)

	a / Å	b / Å	c / Å	V / Å^3	ρ / a.Å^{-3}	Mn-O ax. / Å	Mn-O eq. / Å
**B3LYP**	5.739	5.750	5.739	133.9	0.0299	1.918	1.917
**HSE06**	5.673	5.685	5.673	129.4	0.0309	1.902	1.901
**PBE**	5.722	5.747	5.722	133.1	0.0301	1.921	1.922
**PBE0**	5.668	5.680	5.668	129.0	0.0310	1.900	1.899
**B3LYP-D3**	5.674	5.687	5.674	129.4	0.0309	1.907	1.907
**HSE06-D3**	5.633	5.647	5.633	126.7	0.0316	1.895	1.894
**PBE-D3**	5.678	5.704	5.678	130.0	0.0308	1.914	1.916
**PBE0-D3**	5.630	5.644	5.630	126.5	0.0316	1.894	1.893
**Expt.**[91]	5.670	5.670	5.670	130.9	0.0306	1.916	1.916

α-MnO_2

Table S14: Full structural information for α-MnO_2 from the geometry optimisations performed in this study with all evaluated functionals and their D3-corrected counterparts including: lattice parameters in Å(a, b, c), equilibrium volume in Å^3 (V), atomic density in a.Å^{-3} (ρ) and axial/equatorial Mn-O bond lengths in Å(Mn-O ax. and Mn-O eq.)

	a **/ Å**	***b*** **/ Å**	***c*** **/ Å**	**V / Å^3**	ρ **/ a.Å^{-3}**	**Mn-O ax$_1$ / Å**	**Mn-O ax$_2$ / Å**	**Mn-O eq$_{1/2}$ / Å**	**MN-O eq$_{3/4}$ / Å**
**B3LYP**	9.823	9.823	2.881	278.0	0.0288	1.903	1.921	1.909	1.896
**HSE06**	9.718	9.718	2.856	269.7	0.0297	1.886	1.903	1.878	1.895
**PBE**	9.816	9.816	2.879	277.4	0.0288	1.886	1.934	1.903	1.912
**PBE0**	9.715	9.715	2.853	269.3	0.0297	1.888	1.900	1.876	1.894
**B3LYP-D3**	9.732	9.732	2.867	271.5	0.0295	1.883	1.915	1.886	1.903
**HSE06-D3**	9.661	9.661	2.848	265.8	0.0301	1.875	1.899	1.872	1.891
**PBE-D3**	9.760	9.760	2.870	273.4	0.0293	1.876	1.930	1.897	1.908
**PBE0-D3**	9.663	9.663	2.845	265.7	0.0301	1.877	1.897	1.871	1.890
**Expt.**[88]	9.815	9.815	2.847	274.3	0.0292	1.882	1.901	1.887	1.896

R-MnO_2

Table S15: Full structural information for R-MnO_2 from the geometry optimisations performed in this study with all evaluated functionals and their D3-corrected counterparts including: lattice parameters in Å(a, b, c), equilibrium volume in Å^3 (V), atomic density in a.Å^{-3} (ρ) and axial/equatorial Mn-O bond lengths in Å(Mn-O ax. and Mn-O eq.)

	a **/ Å**	***b*** **/ Å**	***c*** **/ Å**	**V / Å^3**	ρ **/ a.Å^{-3}**	**Mn-O ax$_1$ / Å**	**Mn-O ax$_2$ / Å**	**Mn-O eq$_{1/2}$ / Å**	**MN-O eq$_{3/4}$ / Å**
**B3LYP**	2.884	4.630	9.257	123.6	0.0324	1.903	1.921	1.899	1.907
**HSE06**	2.861	4.564	9.175	119.8	0.0334	1.888	1.902	1.881	1.894
**PBE**	2.878	4.561	9.355	122.8	0.0326	1.888	1.933	1.907	1.911
**PBE0**	2.858	4.568	9.158	119.6	0.0334	1.888	1.900	1.879	1.893
**B3LYP-D3**	2.868	4.427	9.344	118.6	0.0337	1.882	1.914	1.888	1.901
**HSE06-D3**	2.851	4.467	9.198	117.1	0.0341	1.875	1.898	1.874	1.890
**PBE-D3**	2.868	4.457	9.377	119.9	0.0334	1.878	1.927	1.899	1.908
**PBE0-D3**	2.848	4.435	9.242	116.7	0.0343	1.875	1.898	1.874	1.888
**Expt.**[70]	2.866	4.533	9.270	118.5	0.0338	1.889	1.910	1.862	1.923

ZnO

- The experimental structures for the CZB and CsCl-type polymorphs were not obtained at standard conditions.

Hexagonal Wurtzite

Table S16: Full structural information for Hexagonal Wurtzite ZnO from the geometry optimisations performed in this study with all evaluated functionals and their D3-corrected counterparts including: lattice parameters in Å(a, b, c), equilibrium volume in $Å^3$ (V), atomic density in a.$Å^{-3}$ (ρ) and axial/equatorial Zn-O bond lengths in Å(Zn-O ax. and Zn-O eq.)

	a **/ Å**	***b*** **/ Å**	***c*** **/ Å**	**V / $Å^3$**	ρ **/ a.$Å^{-3}$**	**Zn-O tet. / Å**
**B3LYP**	3.284	3.284	5.273	49.3	0.0399	1.997
**HSE06**	3.260	3.260	5.217	48.0	0.0409	1.981
**PBE**	3.284	3.284	5.275	49.3	0.0399	1.998
**PBE0**	3.259	3.259	5.215	48.0	0.0410	1.980
**B3LYP-D3**	3.243	3.243	5.214	47.5	0.0414	1.973
**HSE06-D3**	3.236	3.236	5.175	46.9	0.0419	1.965
**PBE-D3**	3.261	3.261	5.239	48.3	0.0407	1.984
**PBE0-D3**	3.236	3.236	5.177	47.0	0.0418	1.980
**Expt.**[100]	3.249	3.249	5.204	47.6	0.0420	1.974

Cubic Zinc-Blende

Table S17: Full structural information for Cubic Zinc-Blende ZnO from the geometry optimisations performed in this study with all evaluated functionals and their D3-corrected counterparts including: lattice parameters in Å(a, b, c), equilibrium volume in $Å^3$ (V), atomic density in a.$Å^{-3}$ (ρ) and axial/equatorial Zn-O bond lengths in Å(Zn-O ax. and Zn-O eq.)

	a **/ Å**	***b*** **/ Å**	***c*** **/ Å**	**V / $Å^3$**	ρ **/ a.$Å^{-3}$**	**Zn-O tet. / Å**
**B3LYP**	4.616	4.616	4.616	98.4	0.0399	1.999
**HSE06**	4.579	4.579	4.579	96.0	0.0409	1.983
**PBE**	4.615	4.615	4.615	98.3	0.0400	1.998
**PBE0**	4.577	4.577	4.577	95.9	0.0410	1.982
**B3LYP-D3**	4.561	4.561	4.561	94.9	0.0414	1.975
**HSE06-D3**	4.544	4.544	4.544	93.8	0.0419	1.968
**PBE-D3**	4.585	4.585	4.585	96.4	0.0407	1.986
**PBE0-D3**	4.544	4.544	4.544	93.8	0.0419	1.968
**Expt.**[101]	5.434	5.434	5.434	160.5	0.0421	2.353

Rocksalt-type

Table S18: Full structural information for Rocksalt-type ZnO from the geometry optimisations performed in this study with all evaluated functionals and their D3-corrected counterparts including: lattice parameters in Å(a, b, c), equilibrium volume in $Å^3$ (V), atomic density in a.$Å^{-3}$ (ρ) and axial/equatorial Zn-O bond lengths in Å(Zn-O ax. and Zn-O eq.)

	a / Å	***b*** / Å	***c*** / Å	**V** / $Å^3$	ρ / a.$Å^{-3}$	**Zn-O ax./eq.** / Å
**B3LYP**	4.315	4.315	4.3147	98.4	0.0489	2.157
**HSE06**	4.273	4.273	4.2726	96.0	0.0504	2.136
**PBE**	4.319	4.319	4.3186	98.3	0.0488	2.159
**PBE0**	4.268	4.268	4.2677	95.9	0.0505	2.134
**B3LYP-D3**	4.253	4.253	4.2531	94.9	0.0511	2.127
**HSE06-D3**	4.234	4.234	4.2345	93.8	0.0517	2.117
**PBE-D3**	4.283	4.283	4.2832	96.4	0.0500	2.142
**PBE0-D3**	4.232	4.232	4.2321	93.8	0.0518	2.116
**Expt.**[102]	4.270	4.270	4.2700	160.5	0.0505	2.135

CsCl-type

Table S19: Full structural information for CsCl-type ZnO from the geometry optimisations performed in this study with all evaluated functionals and their D3-corrected counterparts including: lattice parameters in Å(a, b, c), equilibrium volume in $Å^3$ (V), atomic density in a.$Å^{-3}$ (ρ) and axial/equatorial Zn-O bond lengths in Å(Zn-O ax. and Zn-O eq.)

	a / Å	***b*** / Å	***c*** / Å	**V** / $Å^3$	ρ / a.$Å^{-3}$	**Zn-O cub.** / Å
**B3LYP**	2.677	2.6766	2.6766	19.2	0.0512	2.318
**HSE06**	2.646	2.6457	2.6457	18.5	0.0530	2.291
**PBE**	2.677	2.6773	2.6773	19.2	0.0512	2.319
**PBE0**	2.641	2.6407	2.6407	18.4	0.0533	2.287
**B3LYP-D3**	2.633	2.6333	2.6333	18.3	0.0538	2.281
**HSE06-D3**	2.620	2.6201	2.6201	18.0	0.0546	2.269
**PBE-D3**	2.656	2.6559	2.6559	18.7	0.0524	2.300
**PBE0-D3**	2.617	2.6166	2.6166	17.9	0.0548	2.266
**Expt.**[103]	2.680	2.6800	2.6800	19.2	0.0510	2.321

ΔE_{D3} vs. ρ plots

TiO_2

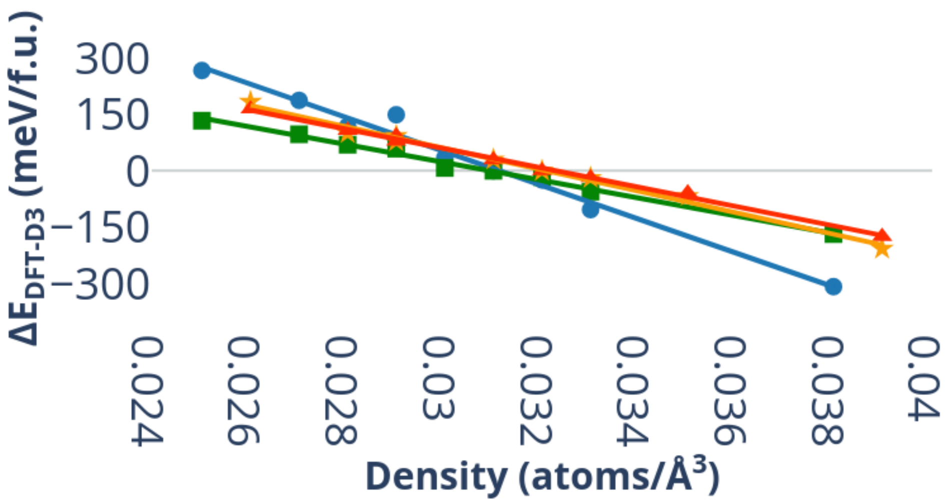

Figure S3: Energetic contribution from the DFT-D3 correction, ΔE_{D3} in meV/f.u. TiO_2 plotted against the atomic density in atoms per cubic Ångström (a.$\AA^{-3}$) of each TiO_2 polymorph for all functionals used in this study. The data colouring is as follows: Blue for B3LYP, orange for HSE06, green for PBE and red for PBE0

MnO_2

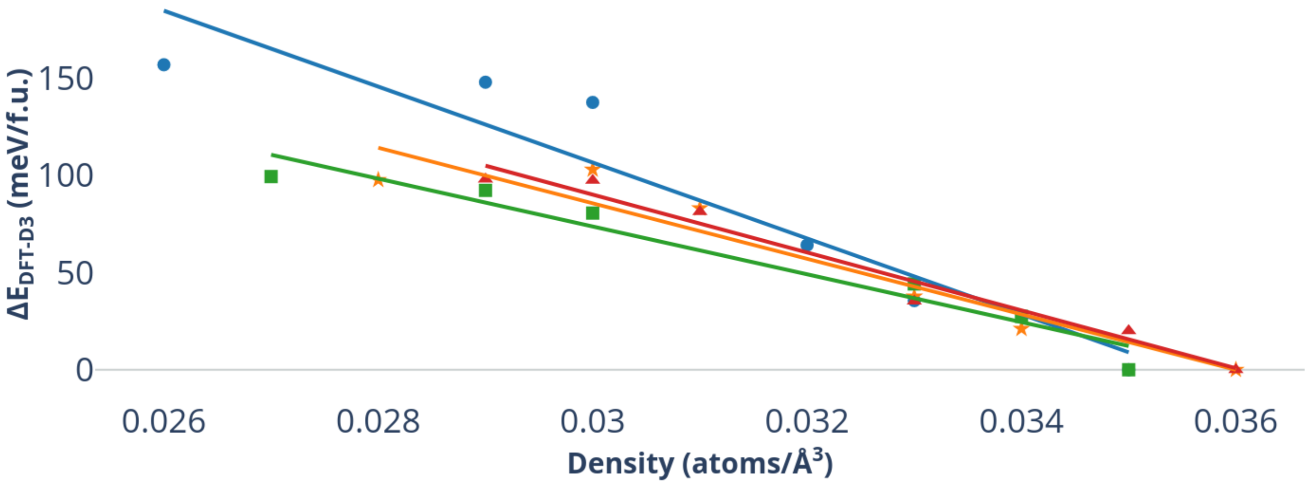

Figure S4: The energetic contribution from the DFT-D3 correction, ΔE_{D3} in meV/f.u. MnO_2 is plotted against the atomic density in atoms per cubic Ångström (a.Å^{-3}) of each TiO_2 polymorph for the representative functionals, PBE and B3LYP. The data colouring is as follows: Blue for B3LYP, orange for HSE06, green for PBE and red for PBE0

ZnO

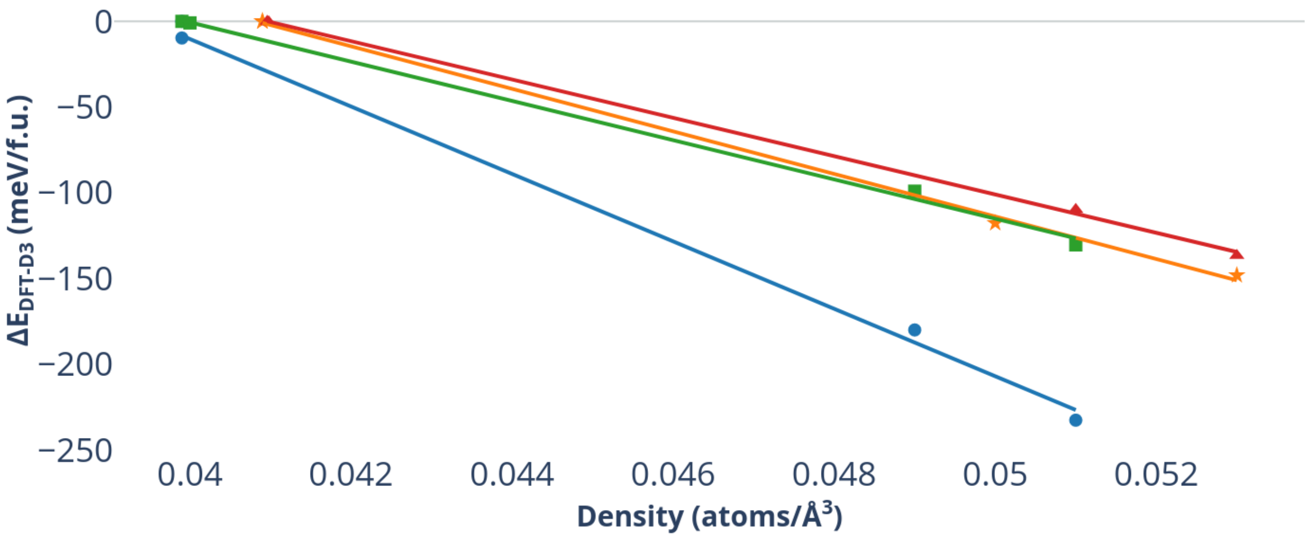

Figure S5: The energetic contribution from the DFT-D3 correction, ΔE_{D3} in meV/f.u. SnO is plotted against the atomic density in atoms per cubic Ångström (a.Å^{-3}) of each TiO_2 polymorph for the representative functionals, PBE and B3LYP. The data colouring is as follows: Blue for B3LYP, orange for HSE06, green for PBE and red for PBE0
